# Yoga for schizophrenia: a systematic review and meta-analysis

**DOI:** 10.1186/1471-244X-13-32

**Published:** 2013-01-18

**Authors:** Holger Cramer, Romy Lauche, Petra Klose, Jost Langhorst, Gustav Dobos

**Affiliations:** 1 Department of Internal and Integrative Medicine, Kliniken Essen-Mitte, Faculty of Medicine, University of Duisburg-Essen, Am Deimelsberg 34a, Essen 45276, Germany

**Keywords:** Schizophrenia, Yoga, Complementary therapies, Meta-analysis, Review

## Abstract

**Background:**

The aim of this review was to systematically review and meta-analyze the effects of yoga on symptoms of schizophrenia, quality of life, function, and hospitalization in patients with schizophrenia.

**Methods:**

MEDLINE/Pubmed, Scopus, the Cochrane Library, PsycInfo, and IndMED were screened through August 2012. Randomized controlled trials (RCTs) comparing yoga to usual care or non-pharmacological interventions were analyzed when they assessed symptoms or quality of life in patients with schizophrenia. Cognitive function, social function, hospitalization, and safety were defined as secondary outcomes. Risk of bias was assessed using the risk of bias tool recommended by the Cochrane Back Review Group. Standardized mean differences (SMD) and 95% confidence intervals (CI) were calculated.

**Results:**

Five RCTs with a total of 337 patients were included; 2 RCTs had low risk of bias. Two RCTs compared yoga to usual care; 1 RCT compared yoga to exercise; and 2 3-arm RCTs compared yoga to usual care and exercise. No evidence was found for short-term effects of yoga compared to usual care on positive symptoms (SMD = -0.58; 95% CI -1.52 to 0.37; P = 0.23), or negative symptoms (SMD = -0.59; 95% CI -1.87 to 0.69; P = 0.36). Moderate evidence was found for short-term effects on quality of life compared to usual care (SMD = 2.28; 95% CI 0.42 to 4.14; P = 0.02). These effects were only present in studies with high risk of bias. No evidence was found for short-term effects on social function (SMD = 1.20; 95% CI -0.78 to 3.18; P = 0.23). Comparing yoga to exercise, no evidence was found for short-term effects on positive symptoms (SMD = -0.35; 95% CI -0.75 to 0.05; P = 0.09), negative symptoms (SMD = -0.28; 95% CI -1.42 to 0.86; P = 0.63), quality of life (SMD = 0.17; 95% CI -0.27 to 0.61; P = 0.45), or social function (SMD = 0.20; 95% CI -0.27 to 0.67; P = 0.41). Only 1 RCT reported adverse events.

**Conclusions:**

This systematic review found only moderate evidence for short-term effects of yoga on quality of life. As these effects were not clearly distinguishable from bias and safety of the intervention was unclear, no recommendation can be made regarding yoga as a routine intervention for schizophrenia patients.

## Background

Schizophrenia is a severe mental disorder that manifests itself mainly by positive symptoms (delusions and hallucinations) and negative symptoms (lack of motivation, reduction in spontaneous speech, and social withdrawal) [1,2]. Impaired cognitive function (difficulties in memory, attention, and executive functioning) is a third important symptom cluster [1,2] that is mainly associated with negative symptoms [3]. This psychopathology has substantial impact on quality of life, well-being and social and occupational function [4-6] and thus creates a considerable socioecomic burden [7,8].

While psychopharmaceutics are effective in improving positive symptoms, their effectiveness on negative symptoms is limited [2,9]. About 30% of patients are therapy-refractory [10]. It has recently been demonstrated that physical activity can relieve symptoms and improve function and quality of life in patients with schizophrenia [11,12]. Yoga is a traditional Indian practice that combines physical activity with lifestyle advice and body awareness techniques such as breath control and meditation [13,14]. Yoga is thought to relieve stress and mental symptoms by increased balance of body, thoughts, and emotions [15]. Systematic reviews have shown that yoga can improve mental functioning and quality of life in physical conditions such as cancer [16,17], menopause [18], and pain [19,20]. As well, yoga has been shown to improve mental disorders such as depression [21], and anxiety disorders [22]. While a prior systematic review suggests effectiveness of yoga in relieving symptoms and improving well-being in patients with schizophrenia [23], no meta-analysis is available.

Therefore, the aim of this review was to systematically assess and meta-analyze the effectiveness of yoga in patients with schizophrenia.

## Methods

PRISMA guidelines for systematic reviews and meta-analyses [24] and the recommendations of the Cochrane Collaboration [25] were followed.

### Eligibility criteria

#### Types of studies

Randomized controlled trials (RCTs) and randomized cross-over studies (only data from the first active treatment phase were used) were eligible. No language restrictions were applied. Studies were eligible only if they were published as full paper.

#### Types of participants

Adults with schizophrenia were eligible if they were diagnosed by

1. The Diagnostic and Statistical Manual (DSM) [1], the Research Diagnostic Criteria (RDC) [26], or the International Classification of Disease (ICD) [27];

2. Any other clinician-based diagnosis criterion.

3. Unclear diagnostic criteria but were currently treated for schizophrenia.

Studies involving participants with comorbid physical or mental disorders were eligible for inclusion.

#### Types of interventions

##### Experimental

Yoga interventions including at least 1 of the following: physical activity, breath control, meditation, and/or lifestyle advice (based on yoga theory and/or traditional yoga practices) were eligible. No restrictions were made regarding yoga tradition, length, frequency or duration of the program. Studies on multimodal interventions, such as mindfulness-based stress reduction and mindfulness-based cognitive therapy (that include yoga amongst others) [28] were excluded. Co-interventions were allowed.

##### Control

1. Usual care or standard care.

2. Exercise or other active non-pharmacological interventions.

#### Types of outcome measures

To be eligible, RCTs had to assess at least 1 primary outcome:

1. Improvement in the severity of symptoms of schizophrenia, measured by clinician-rated scales, such as the Brief Psychiatric Rating Scale (BPRS) [29], the Positive and Negative Syndrome Scale (PANSS) [30], the Clinical Global Impression Scale (CGI) [31], or any other validated scale.

2. Improvement in quality of life or well-being measured by any validated scale such as the WHO Quality of Life-BREF quality of life assessment [32].

Secondary outcomes included:

1. Improvement in cognitive function, measured by test batteries such as the NIMH Measurement and Treatment Research to Improve Cognition in Schizophrenia (MATRICS) [33] or any other validated battery.

2. Social function, measured by any validated scale such as the Socio-Occupational Functioning Scale (SOFS) [34].

3. Hospitalization, assessed as e.g. number of admissions or days in hospital in a pre-defined follow-up period [35].

4. Safety of the intervention assessed as amount of extrapyramidal symptoms or number of adverse events.

### Search methods

Medline/PubMed, Scopus, the Cochrane Library, PsycINFO, and IndMED were searched from their inception through 28 August 2012. The literature search was constructed around search terms for “yoga” and search terms for “schizophrenia”. For PubMed, the following search strategy was used: *(“Yoga”[Mesh] OR “Yoga”[Title/Abstract] OR “Yogic”[Title/Abstract]) AND (“Schizophrenia”[Mesh] OR “Schizophrenia”[Title/Abstract] OR “Schizophrenic”[Title/Abstract])*. The search strategy was adapted for each database as necessary.

Additionally, reference lists of identified original articles or reviews were searched manually.

Abstracts identified during literature search were screened by 2 review authors independently. Potentially eligible articles were read in full by 2 review authors to determine whether they met the eligibility criteria. Disagreements were discussed with a third review author until consensus was reached. If necessary, additional information was obtained from the study authors.

### Data extraction and management

Two authors independently extracted data on patients (e.g. age, diagnosis), methods (e.g. randomization, allocation concealment), interventions (e.g. yoga type, frequency, and duration), control interventions (e.g. type, frequency, duration), co-interventions, outcomes (e.g. outcome measures, assessment time points), and results using an a priori developed data extraction form. Discrepancies were discussed with a third review author until consensus was reached. If necessary, the study authors were contacted for additional information.

### Risk of bias in individual studies

Two authors independently assessed risk of bias using the risk of bias tool proposed by the Cochrane Back Review Group [36]. This tool assesses risk of bias on the following domains: selection bias, performance bias, attrition bias, reporting bias, and detection bias using 12 criteria. Risk of bias was assessed for each criterion as 1) low risk of bias, 2) unclear, 3) high risk of bias. Discrepancies were discussed with a third review author until consensus is reached. Studies that met at least 6 of the 12 criteria and had no serious flaw were rated as having low risk of bias. Studies that met fewer than 6 criteria or had a serious flaw were rated as having high risk of bias [36].

### Data analysis

#### Assessment of effect size

Separate meta-analyses were conducted for

1) Short-term and long-term effects. Short-term outcomes were defined as outcome measures taken closest to 12 weeks after randomization and long-term outcomes as measures taken closest to 12 months after randomization [35].

2) Different control interventions (usual care; exercise).

Meta-analyses were conducted using Review Manager 5 software (Version 5.1, The Nordic Cochrane Centre, Copenhagen) by a random effects model [25]. Meta-analyses were conducted if at least 2 RCTs for a specific comparison were available [25].

Standardized mean differences (SMD) with 95% confidence intervals (CI) were calculated as the difference in means between groups divided by the pooled standard deviation. Where no standard deviations were available, they were calculated from standard errors, confidence intervals or t values [25], or attempts were made to obtain the missing data from the trial authors by email.

Where data were suspected to be skewed, this was tested by subtracting the observed mean from the highest possible value of the respective outcome measure and dividing this by the standard deviation [25]. Ratios below 2 were regarded as indicating possible skewness [25].

A negative SMD was defined to indicate beneficial effects of yoga compared to the control intervention for symptoms and hospitalization while a positive SMD was defined to indicate beneficial effects of yoga compared to the control intervention for well-being and function. If necessary, scores were inverted by subtracting the mean from zero [25].

Cohen’s categories were used to evaluate the magnitude of the overall effect size with 1) SMD = 0.2 to 0.5: small; 2) SMD = 0.5 to 0.8: moderate and 3) SMD > 0.8: large effect sizes [37].

Levels of evidence were determined as 1) strong evidence: consistent findings among multiple RCTs with low risk of bias; 2) moderate evidence: consistent findings among multiple high-risk RCTs and/or one low-risk RCT; 3) limited evidence: one RCT with high risk of bias; 4) conflicting evidence: inconsistent findings among multiple RCTs; and 5) no evidence: no RCTs [38].

### Assessment of heterogeneity

The I^2^ statistics, a measure of how much variance between studies can be attributed to differences between studies rather than chance, was used to analyze statistical heterogeneity between studies. The magnitude of heterogeneity was categorized as 1) I^2^ = 0-25%: low heterogeneity; I^2^ = 26-50%: moderate heterogeneity; I^2^ = 51-75%: substantial heterogeneity; and I^2^ = 76-100%: considerable heterogeneity [25,39]. The Chi^2^ test was used to assess whether differences in results were compatible with chance alone. Given the low power of this test when only few studies or studies with low sample size are included in a meta-analysis, a P-value ≤ 0.10 was regarded to indicate significant heterogeneity [25].

### Subgroup and sensitivity analyses

Subgroup analyses were conducted for type of participants (manual-based diagnosis; other or unclear diagnosis). Further subgroup analyses were conducted for duration of the intervention (less than 12 weeks; more than 12 weeks).

To test the robustness of significant results, sensitivity analyses were conducted for studies with high versus low risk of bias.

If statistical heterogeneity was present in the respective meta-analysis, subgroup and sensitivity analyses were also used to explore possible reasons for heterogeneity.

### Risk of bias across studies

If at least 10 studies were included in a meta-analysis, the study protocol planned to assess publication bias by visual analysis of funnel plots generated using Review Manager 5 software [40]. As less than 10 studies were finally included (see below), publication bias could not be assessed.

## Results

### Literature search

**Figure 1:**
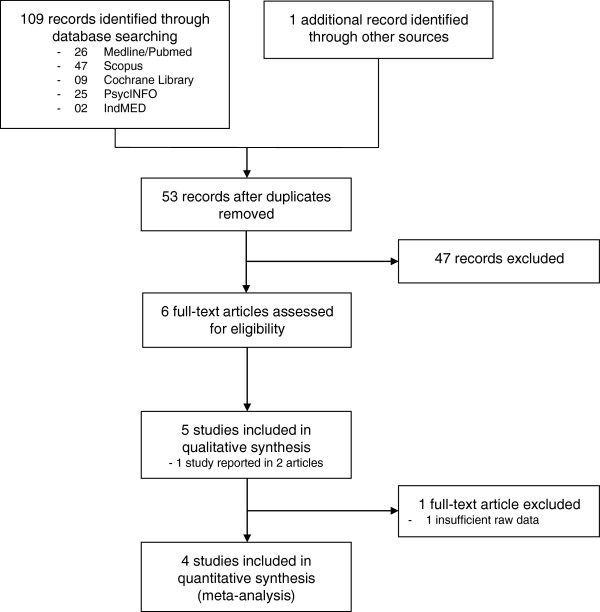
Flowchart of the results of the literature search.

The literature search retrieved 109 records, and 1 additional record was retrieved through other sources. Fifty-three nonduplicate records were screened and 47 records were excluded because they were no RCTs and/or yoga was not an intervention. Six full-text articles reporting on 5 RCTs with a total of 337 patients were included in qualitative analysis [41-46]. One randomized cross-over trial was excluded from quantitative synthesis as data from the first active treatment phase were not reported and could not be retrieved from the trial authors [44]. Finally, 4 studies with 288 patients were included in the meta-analysis (Figure 1).

### Study characteristics

Characteristics of the sample, interventions, outcome assessment and results are shown in Table 1.

**Table 1:** Characteristics of the included studies

**Reference**	**Patients**	**Cointerventions ns**	**Intervention groups**		**Longest follow-up**	**Outcome measures**	**Results**	
	**(N, Diagnosis, Age)**		**(program length, frequency, duration)**					
			**Treatment**	**Control**		1) Symptoms	Short-term	Long-term
						2) Well-being		
						3) Cognitive function		
						4) Social function		
						5) Hospitalization		
						6) Safety		
Behere et al., 2011 [41] Varambally et al., 2012 [42]	119 patients with Schizophrenia according to DSM IV CGI≥3 YOGA 32.8±10.0 y EX 30.6±7.3 y WL 33.6±9.5 y	Antipsychotic drugs	**Yogasana** (YOGA) 4 months: Month 1: 25x45 minutes instructed SVYASA Yoga (loosening exercises, postures, breathing, relaxation, no meditation) Month 2-4: home based training	1. **Exercise** (EX) 4 months Month 1: 25x45 min. instructed physical exercise (walking, jogging, postures, relaxation) Month 2-4: home based training 2. **Usual care** (UC) 4 months	4 months	1) PANSS a) positive symptoms b) negative symptoms c) total 3) TRACS 4) SOFS total 6) Extrapyramidal Symptoms	1) a) YOGA: sign. improvement; EX/WL: n.s. b) YOGA: sign. improvement; EX/WL: n.s. c) YOGA: sign. improvement; EX/WL: n.s. 3) YOGA: sign. improvement; EX/WL: n.s. 4) YOGA: sign. improvement; EX/WL: n.s. 6) n.s.	n.a.
Duraiswamy et al., 2007 [43]	61 patients with Schizophrenia according to DSM IV CGI≥4 YOGA 32.5±7.9 y EX 31.3±7.9 y	Antipsychotic drugs	**Yogasana** (YOGA) 4 months Month 1: 25x45 minutes instructed SVYASA Yoga(loosening exercises, postures, breathing, relaxation, no meditation) Month 2-4: home-based training	1. **Exercise** (EX) 4 months Month 1: 25x45 min. instructed physical exercise (walking, jogging, postures, relaxation) Month 2-4: home-based training	4 months	1) PANSS a) positive symptoms b) negative symptoms c) total 2) WHO QOL-BREF a) physical b) psychological c) social d) environmental 4) SOFS total 6) Simpson Angus Scale for Extrapyramidal Symptoms	1) a) n.s. b) YOGA > EX c) YOGA > EX. 2) a) YOGA > EX b) YOGA > EX c) YOGA > EX d) YOGA > EX 4) YOGA > EX 6) n.s.	n.a.
Vancampfort et al., 2011 [44]	49 patients with schizophrenia or schizoaffective disorder CGI≥4 Women: 32.8±8.9 y Men: 31.8±8.7 y	Hospital inpatient treatment	**Hatha yoga** (YOGA) Single 30-minutes session Bodily postures, coordination, strength, flexibility, balance, breath awareness, relaxation.	1. **Exercise** (EX) Single 20-minutes session Ergometer training 2. **Usual care** (UC) Single 20-minutes session Reading	Immediately	2) SEES	2) YOGA > UC	n.a.
Visceglia et al., 2011 [45]	18 patients with schizophrenia YOGA: 37.4±13.7 y WL: 48.1±11.2 y	Hospital inpatient treatment	**Yoga** (YOGA) 8 weeks: 2 x 45 minutes per week Stretching, movements, breathing, relaxation	**Usual care** (UC) 8 weeks	8 weeks	1) PANSS a) positive symptoms b) negative symptoms c) total 2) WHO QOL-BREF a) physica b) psychological c) environmental 4) WHO QOL-BREF 6) adverse events	1.) a) YOGA > UC b) YOGA > UC c) YOGA > UC 2) a) YOGA > UC b) YOGA > UC c) n.s. 4) n.s. 6) n.s.	n.a.
Xie et al., 2006 [46]	90 patients with schizophrenia according to CCMD-3 YOGA: 28.2±8.3 y UC: 30.5±9.4 y	Antipsychotic drugs	**Yoga** (YOGA) 8 weeks 4-5x60 minutes per week Bodily postures, breathing techniques, meditation, relaxation	**Usual care (UC) 8 weeks**	8 weeks	2) GQOLI-74 a) material life b) physical function c) psychological function 4) GQOLI-74	2) a) n.s. b) YOGA > UC c) YOGA > UC 4) YOGA > UC	n.a.

Abbreviations: > – significant group difference; CCMD Chinese Classification of Mental Disorders, GQOLI-74 General Quality of Life Inventory-74, DSM Diagnostic and Statistical Manual of Mental Disorders, n.a. not assessed, n.s. not significant, PANSS The Positive and Negative Syndrome Scale, SEES Subjective Exercise Experiences Scale, SOFS Socio-Occupational Functioning Scale, TRACS TREND Accuracy Scale, TREND Tool for Recognition of Emotions in Neuropsychiatric Disorders, WHO QOL-BREF WHO Quality of Life-BREF quality of life assessment, y - years.

#### Setting and participant characteristics

Of the 5 RCTs that were included, 3 originated from Asia (2 from India [41-43], and 1 from China [46]), 1 from North America (USA) [45], and 1 from Europe (Belgium) [44]. Patients were recruited from psychiatric outpatient services [41-43], and/or psychiatric inpatient services [42-45].

Patients in 2 RCTs were diagnosed with schizophrenia according to DSM-IV [41-43], and patients in 1 RCTs were diagnosed according to CCD-3 (Chinese Classification of Mental Disorders) [46]. The remaining 2 RCTs included patients that were hospitalized for schizophrenia [44,45] or schizoaffective disorder [44] but did not state the diagnostic criteria used. Patients in 3 RCTs were required to have a clinical global severity scale rating of at least 3 [41,42] or 4 [43,44]. Mean duration of illness ranged from 76.4 months to 129.7 months with a median of 88.6 months. Patients in all RCTs were on stabilized antipsychotic medication. Patients’ mean age ranged from 28.2 years to 48.1 years with a median age of 32.5 years. Between 31.0% and 60.0% (median: 33.3%) of patients in each study were female.

Intervention characteristics

Yoga was based on the yoga module developed by Swami Vivekananda Yoga Anusandhana Samsthana (SVYASA) in 2 RCTs [41-43], and on the principles of hatha yoga in 1 RCT [44]. The remaining 2 RCTs did not state yoga tradition [45,46]. All yoga programs included yoga postures, breath control, and meditation/relaxation. Program length and intensity varied and included: 1 single 30-minute session [44]; 2 45-minute sessions per week over a period of 8 weeks [44]; 4–5 60-minute sessions per week over a period of 8 weeks [46]; and 25 45-minute sessions over a period of 1 months followed by 3 months of home-based yoga [41-43]. Yoga was taught by physicians [45], physical or occupational therapists trained to teach yoga therapy [43,44,46], or certified yoga teachers [41,42].

Two RCT compared yoga to usual care [45,46], and 1 RCT compared yoga to exercise [43]. Two 3-arm RCTs compared yoga to usual care and exercise [41,42,44]. Exercise interventions were matched to the yoga interventions in terms of frequency, length, and duration and included walking, jogging, exercise in standing and sitting postures, and relaxation in 2 RCTs [41-43]. In 1 RCT a single 30-minute yoga session was compared to a single 20-minute ergometer training session [44]. Exercise was taught by (physical) therapists trained to teach exercise therapy [41-44].

Outcome measures

Symptoms of schizophrenia were assessed in 3 RCTs using the Positive and Negative Syndrome Scale [41-43,45]. Quality of life or well-being was assessed in 4 studies using the WHO Quality of Life-BREF quality of life assessment [43,45], the General Quality of Life Inventory-74 [46], or the Subjective Exercise Experiences Scale [44]. Cognitive function was measured by 1 RCT using the Tool for Recognition of Emotions in Neuropsychiatric Disorders [41]. Social function was assessed in 4 RCTs using the Socio-Occupational Functioning Scale [41-43], the WHO Quality of Life-BREF quality of life assessment [45], or the General Quality of Life Inventory-74 [46]. No study reported data on hospitalization. Safety was assessed as number of adverse events in 1 RCT [45] and as amount of extrapyramidal symptoms in 2 RCTs [41-43].

While all RCTs reported short-term effects, no RCT reported long-term effects.

#### Risk of bias in individual studies

Two RCTs had low risk of bias [41-43], and 3 RCTs had high risk of bias [44-46] (Table 2). Risk of selection bias was mixed; all RCTs reported adequate randomization, while only 1 RCT reported adequate allocation concealment [41,42]. No RCT reported blinding of participants or providers, but 3 RCTs reported adequate blinding of outcome assessors [41-43,45]. Co-interventions were adequately reported and comparable between groups in 3 RCTs [41-43,46]. Attrition bias was high in all studies as no RCT had an acceptable and described drop-out rate and no RCT used an intention-to-treat analysis. Risk of selective outcome reporting was high in 1 study that reported different outcomes from the same RCT in multiple publications without disclosing the entire study protocol [41,42].

**Table 2:** Risk of bias of the included studies

	**Selection bias:**			**Performance bias:**				**Attrition bias:**		**Reporting bias:**	**Detection bias:**		
**Author, year**	**Adequate random sequence generation**	**Adequate allocation concealment**	**Similar baseline characteristics**	**Adequate participant blinding**	**Adequate provider blinding**	**Similar or no co-interventions**	**Acceptable compliance**	**Acceptable and described drop-out rate**	**Inclusion of an intention-to-treat analysis**	**No selective outcome reporting**	**Adequate outcome assessor blinding**	**Similar timing of outcome assessment**	**Total: (max. 12)**^**a**^
Behere et al., 2011 [41] Varambally et al., 2012 [42]	Yes	Yes	Yes	No	No	Yes	Unclear	No	No	Unclear	Yes	Yes	6
Duraiswamy et al., 2007 [43]	Yes	Unclear	Yes	No	Unclear	Yes	Unclear	No	No	Yes	Yes	Yes	6
Vancamport et al., 2011 [44]	Yes	Unclear	Unclear	Unclear	Unclear	Unclear	Yes	No	No	Yes	Unclear	Yes	4
Visceglia et al., 2011 [45]	Yes	Unclear	Yes	No	Unclear	Unclear	Unclear	Unclear	Unclear	Yes	Yes	Yes	5
Xie et al., 2006 [46]	Yes	Unclear	Yes	No	Unclear	Yes	Unclear	No	No	Yes	Unclear	Yes	5

^a^Higher scores indicate lower risk of bias.

#### Outcomes

One RCT did not report means and SDs due to the skewness of the baseline data [42]. Post-intervention means and SDs were provided by the trial authors on request. These data were tested for skewness and there was no evidence for skewness.

#### Yoga vs. usual care

**Figure 2:**
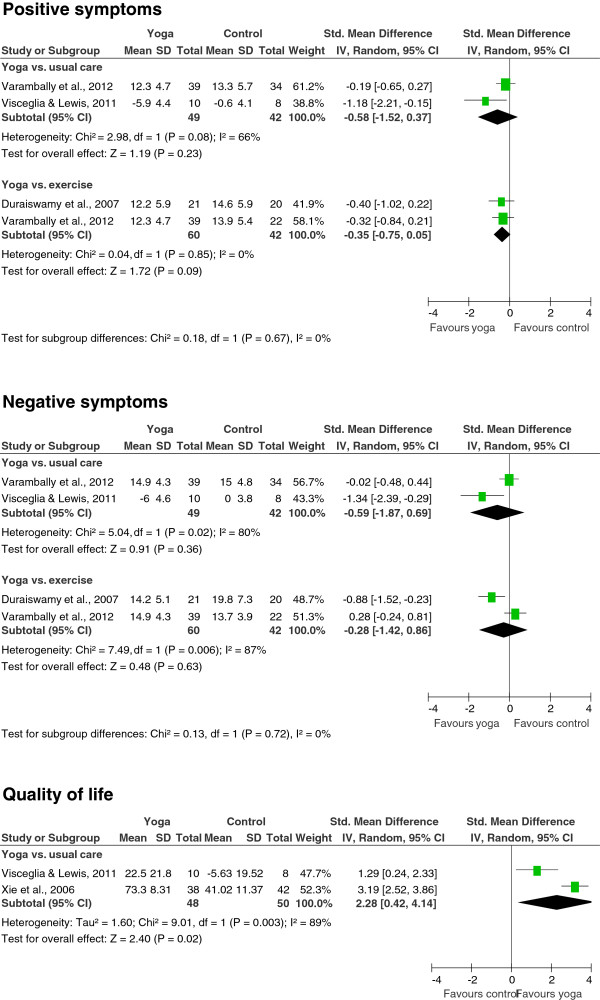
Effects of yoga compared to usual care and exercise on primary outcomes: positive symptoms, negative symptoms, and quality of life.

**Figure 3:**
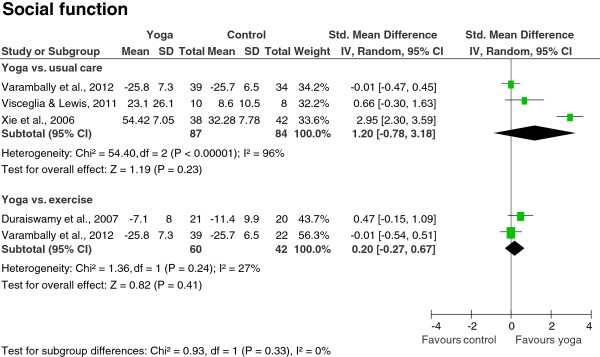
Effects of yoga compared to usual care and exercise on secondary outcomes: social function.

Meta-analyses revealed no evidence for short-term effects of yoga on positive symptoms (SMD = -0.58; 95% CI -1.52 to 0.37; P = 0.23; heterogeneity: I^2^ = 66%; Chi^2^ = 2.98; P = 0.08) or negative symptoms (SMD = -0.59; 95% CI -1.87 to 0.69; P = 0.36; heterogeneity: I^2^ = 80%; Chi^2^ = 5.04; P = 0.02) compared to usual care (Figure 2). There was moderate evidence for a large short-term improvement of quality of life in the yoga groups compared to usual care (SMD = 2.28; 95% CI 0.42 to 4.14; P = 0.02; heterogeneity: I^2^ = 89%; Chi^2^ = 9.01; P < 0.01) (Figure 2). Only 1 RCT assessed cognitive function [41] and there was no evidence for group differences in this RCT (SMD = 0.08; 95% CI -0.49 to 0.64). No evidence was found for short-term effects on social function (SMD = 1.20; 95% CI -0.78 to 3.18; P = 0.23; heterogeneity: I^2^ = 96%; Chi^2^ = 54.40; P < 0.01) (Figure 3).

#### Yoga vs. exercise

When comparing yoga to exercise, no evidence was found for short-term effects on positive symptoms (SMD = -0.35; 95% CI -0.75 to 0.05; P = 0.09; heterogeneity: I^2^ = 0%; Chi^2^ = 0.04; P = 0.85), or negative symptoms (SMD = -0.28; 95% CI -1.42 to 0.86; P = 0.63; heterogeneity: I^2^ = 87%; Chi^2^ = 7.49; P < 0.01) (Figure 2). One RCT assessed quality of life [43] and there was no evidence for group differences in this RCT (SMD = 0.17; 95% CI -0.27 to 0.61). Similarly, only 1 RCT assessed cognitive function [41] and there was no evidence for group differences (SMD = 0.14; 95% CI -0.47 to 0.74). No evidence was found for short-term effects on social function (SMD = 0.20; 95% CI -0.27 to 0.67; P = 0.41; heterogeneity: I^2^ = 27%; Chi^2^ = 1.36; P = 0.24) (Figure 3).

#### Safety

Safety data were reported in 3 RCTs. One RCT reported adverse events and reported that no serious adverse events were observed [45]. Two RCT assessed extrapyramidal symptoms and found no differences between yoga and usual care [41,42] or exercise [41-43].

#### Subgroup analyses

One study with unclear method of diagnosis [45] found limited evidence for effects on positive and negative symptoms when comparing yoga to usual care. These group differences were absent in studies with manual-based diagnosis [41,42].

One study that used a short (less than 3 weeks) yoga intervention [45] found limited evidence for effects on positive and negative symptoms when comparing yoga to usual care. These group differences were absent in studies that used long (more than 3 weeks) yoga interventions [41,42].

#### Sensitivity analyses

Sensitivity analyses demonstrated a significant effect on quality of life in studies with high risk of bias comparing yoga to usual care [45,46] whereas no studies with low risk of bias where available for this comparison.

#### Risk of bias across studies

As less than 10 studies were included in each meta-analysis, funnel plots were not analyzed.

## Discussion

This systematic review found moderate evidence for short-term improvements of quality of life in schizophrenia patients after yoga interventions. Only limited evidence was found for symptom relief and this evidence was based on only 1 study with unclear diagnostic method and short yoga intervention. No evidence was found for improved function. Safety data were only insufficiently reported.

The results of this review are not in line with those of previous qualitative reviews on yoga for schizophrenia. Vancampfort et al. [23] included 3 RCTs that were also included in the present meta-analysis and concluded that yoga can be a useful add-on treatment to reduce psychopathology in schizophrenia. As these conclusions were based on the results that were reported in the original articles, and only 2 out of 3 RCTs reported post-intervention group comparisons, these results were not robust against reporting bias. Another review that included 4 RCTs that were also included in the present review concluded that the prescription of yoga for schizophrenia was evidence-based [47]. A third review that included also unpublished and uncontrolled studies concluded that yoga has been demonstrated to be feasible and effective as an add-on treatment in schizophrenia [48]. None of these reviews included a meta-analysis.

The limited evidence that shorter yoga interventions might be more effective than longer ones is in line with the results of meta-analyses on yoga for pain [19] or fatigue [49].

### External and internal validity

Patients in the included studies were recruited from psychiatric inpatient and outpatient services in North America, Europe, and Asia. Patients diagnosed by different manuals were included. All patients were treated with antipsychotic medication. The majority of patients were males and in the reproductive age range and were diagnosed on average 6 to 11 years ago. The results of this review are therefore applicable to the vast majority of schizophrenia patients in clinical practice. They might however be less applicable to newly diagnosed schizophrenia patients.

Three out of 5 studies had high risk of bias [44-46], one of them was not included in the meta-analysis. Only 1 study reported adequate allocation concealment [41,42]; and only 3 studies reported adequate blinding of outcome assessors [41-43,45]. No study had acceptable drop-out rates or applied an intention-to-treat analysis. The only evidence of effectiveness that was revealed in meta-analysis – the effect on quality of life when comparing yoga to usual care – was present only in studies with high risk of bias. Therefore, this effect cannot be regarded as robust against potential methodological biases.

### Strengths and weaknesses

This is the first meta-analysis available on yoga for schizophrenia. Besides psychopathology, patient-centered outcomes were used [35]. No language restrictions were imposed; thereby this work could include more RCTs than prior reviews [23,48].

The primary limitation of this review is the small total number of eligible RCTs. Therefore, the effect size estimates derived in the meta-analyses were highly unstable. The overall high risk of bias further restricts the interpretation of the results and publication bias could not be ruled out. As no RCT reported longer-term effects, the results of this review are only applicable to short-term effects. Furthermore, means and SDs were not included in the original publication of 1 RCT [42]. While data were published for a subsample of this RCT [41], we decided to use data that were provided by the trial authors on request. While this approach improved the power of the analysis, it might also have introduced bias. Co-interventions were not clearly described in all RCTs; safety data were insufficiently reported; and no RCT reported adherence rates. While the yoga intervention itself was comparable in all RCTs, the intensity of the program varied.

### Implications for further research

Given the low number of available studies, definite conclusions about the effectiveness of yoga in patients with schizophrenia cannot be drawn at the moment. Future studies should ensure rigorous methodology and reporting, mainly adequate sample size, adequate randomization, allocation concealment, intention-to treat analysis, and blinding of at least outcome assessors [50]. Yoga often involves physical exercise and/or meditation or mindfulness practice [13,14]. As exercise interventions have shown effectiveness in improving schizophrenia psychopathology [11], and mindfulness-based interventions have shown preliminary but promising effects in patients with psychosis [51,52], dismantling studies that separately evaluate the effects of different components of yoga seem warranted. As yoga has been shown to promote body awareness [15] which seems to be impaired in schizophrenia patients [53], it might be worthwhile to investigate the effects of yoga on body awareness in schizophrenia.

## Conclusions

This systematic review found only moderate evidence for short-term effects of yoga on quality of life. As these effects were not clearly distinguishable from bias and safety of the intervention remains unclear in this patient population, no recommendation can be made regarding yoga as a routine intervention for schizophrenia patients.

## Competing interests

All authors declare that they have no competing interests.

## Authors’ contributions

HC was responsible for conception and design of the review, carried out the literature search, performed data extraction, data analysis, and assessment of risk of bias, and drafted the manuscript. RL and PK performed data extraction and assessment of risk of bias, and critically revised the manuscript. JL and GD critically revised the manuscript. All authors read and approved the final manuscript.

## Pre-publication history

The pre-publication history for this paper can be accessed here:

http://www.biomedcentral.com/1471-244X/13/32/prepub
